# Fluorescence-guided Two-port Robotic Gastrectomy Versus Conventional Laparoscopic Gastrectomy: A Nonrandomized Controlled Trial

**DOI:** 10.1097/AS9.0000000000000318

**Published:** 2023-07-26

**Authors:** Seohee Choi, Na Young Kim, Youn Nam Kim, Sung Hyun Park, Ki-Yoon Kim, Minah Cho, Yoo Min Kim, Woo Jin Hyung, Hyoung-Il Kim

**Affiliations:** From the * Department of Surgery, National Health Insurance Service Ilsan Hospital, Goyang, Republic of Korea; † Department of Anesthesiology, Yonsei University College of Medicine, Seoul, Republic of Korea; ‡ Department of Biostatistics, Anne Consulting, Seoul, Republic of Korea; § Department of Surgery, Yonsei University College of Medicine, Seoul, Republic of Korea; ∥ Gastric Cancer Center, Yonsei Cancer Center, Yonsei University Health System, Seoul, Republic of Korea; ¶ Open NBI Convergence Technology Research Laboratory, Severance Hospital, Yonsei University Health System, Seoul, Republic of Korea.

**Keywords:** gastric cancer, robotic surgery, laparoscopic surgery, two-port surgery, image-guided surgery

## Abstract

**Objective:**

To compare the number of retrieved lymph nodes between conventional laparoscopic gastrectomy (CLG) and robotic gastrectomy integrated with fluorescence guidance and a two-port system (integrated robotic gastrectomy, IRG).

**Background:**

The benefits of robotic surgery over laparoscopic surgery for gastric cancer have not yet been established. Using built-in features of robotic system, further benefit can be provided to the patients with effective lymphadenectomy and enhanced recovery.

**Methods:**

A nonrandomized controlled trial was performed by a single surgeon at single-center, tertiary referral hospital between January 2018 and October 2021. Overall, 140 patients scheduled to undergo minimally invasive subtotal gastrectomy for early gastric cancer were enrolled. The primary endpoint was the number of retrieved lymph nodes. Secondary endpoints were complications, hospital stay, pain score, body image, and operative cost.

**Results:**

This study analyzed 124 patients in the per-protocol group (IRG, 64; CLG, 60). The number of retrieved lymph nodes was higher in the IRG group than those in the CLG group (IRG vs CLG; 42.1 ± 17.9 vs 35.1 ± 14.6, *P* = 0.019). Moreover, other surgical parameters, such as hospital stay (4.1 ± 1.0 vs 5.2 ± 1.8, *P* < 0.001) and body image scale (better in 4 of the 10 questions), were significantly better in the IRG than in the CLG.

**Conclusions:**

Robotic surgical procedures integrated with fluorescence guidance and a reduced-port system yielded more retrieved lymph nodes. In addition, the IRG group showed better perioperative surgical outcomes, particularly regarding the length of hospital stay and postoperative body image.

**Trial registration:**

NCT03396354

## Abstract 2

Mini-Abstract: In this nonrandomized controlled trial to determine the advantage of robotic gastrectomy integrated with Single-site^®^ and Firefly over conventional laparoscopic gastrectomy, fluorescence-guided resection yielded a higher number of retrieved lymph nodes, and two-port surgery contributed to a shorter hospital stay and better postoperative body image.

Minimally invasive surgery has rapidly penetrated the era of gastrointestinal surgery and is currently a standard modern surgical technique. Expecting better operative performance by overcoming the limitations of laparoscopy, surgeons have performed robotic gastrectomy for gastric cancer.^1,2^ It has the additional features of a console system and wristed instrumentation, which are expected to facilitate better surgical outcomes.^2^ However, a better surgical outcome with the robot was observed only in prostate and rectal cancer.^3–5^ In gastric cancer surgery, since the initial case series report,^6^ slow but steady accumulation of evidence has been in progress.^7–10^ Although a few reports have shown a significantly lower complication rate than that of the historical control of laparoscopy,^11,12^ other reports have revealed the minimal benefit of robotic gastrectomy in surgical outcomes compared with that of laparoscopy.^7,8,13^

Therefore, we used two additional built-in features during the conventional robotic surgery to take full advantage of the robotic system. Fluorescence-guided surgery (Firefly) can improve the effectiveness of lymphadenectomy because it visualizes the lymphatic channel.^14^ Furthermore, pancreatic injury during lymphadenectomy would be reduced when fluorescence can differentiate lymph nodes from pancreatic tissue. Conversely, two-port gastrectomy is possible if the surgeon uses the Single-site system.^15^ Furthermore, these two features are not mutually exclusive, and the surgeon can use them simultaneously.

Hypothetically, integrating fluorescence-guided lymphadenectomy and two-port robotic gastrectomy (IRG) may provide better outcomes in gastric cancer surgery. Notably, this procedure was applied in clinical practice after completing the initial phase I/II clinical trial to determine its feasibility and safety.^15^ Moreover, after accumulating experience to overcome the learning effect, surgical outcomes of the IRG also showed better surgical outcomes than those of the conventional laparoscopic gastrectomy (CLG).^16^ Therefore, a prospective nonrandomized controlled clinical trial was designed and conducted to validate this finding.

## METHODS

### Trial Design

This was a nonrandomized, controlled group clinical trial performed by a single surgeon at single-center between January 2018 and October 2021 that compared surgical outcomes following robotic gastrectomy integrated with Single-site and Firefly features with those of the CLG. The study was conducted per the ethics of the Declaration of Helsinki and was approved by the Institutional Review Board of Severance Hospital (4-2017-1066). In addition, written informed consent was obtained from all patients. This study was registered at ClinicalTrials.gov (NCT03396354). This trial was supported through a research grant provided by Intuitive Surgical Inc.

### Participants and Allocation

All consecutive patients with early gastric cancer (EGC) scheduled for minimally invasive distal gastrectomy were screened for inclusion during the study period. The exclusion criteria were as follows: cancer requiring total gastrectomy or major combined resection, metastatic or nonresectable lesions, other active cancer histories, pregnant women, and high risk of major adverse cardiovascular events, which was identified by cardiologist. To perform a practically applicable clinical trial, we conducted this study as a nonrandomized prospective cohort study. All patients who underwent robotic distal gastrectomy for EGC were asked to participate in this study. After an enrolled patient underwent robotic gastrectomy, patients of the same sex and similar age undergoing laparoscopic distal gastrectomy were included in the control group upon acceptance of enrollment. The screening was repeated until the pair were matched for the study.

### Interventions

Detailed information on integrated robotic gastrectomy has been described in previous reports.^16^

#### da Vinci

The version of da Vinci (Intuitive Surgical, Sunnyvale, CA) used in this study was Xi or Si, in which Single-Site and Firefly were built-in.

#### Endoscopic ICG injection

The patient underwent an endoscopic injection of indocyanine green (ICG, Dongindang Pharmaceutical) 1 day before surgery. In total, 1.5-mg ICG was injected into four quadrants of the tumor mass.

#### Single-site

During the operation, the patient was positioned supine, a 2-cm incision on the umbilicus was performed, and a port for a Single-site system was applied to the wound. Two robotic arms, one scope, and one assistant port were placed in the gel port for surgery using a Single-site system. Subsequently, a separate incision for a 12-mm trocar was made on the right flank of the patient. This port was used to insert a harmonic scalpel during dissection and a stapler during reconstruction.

#### Surgery

The lymph node dissection and reconstruction procedures were similar in the robotic and laparoscopic surgeries, respectively, as previously reported.^14,17^ Beginning with omentectomy, clearing of infraduodenal lymph nodes, transection of the duodenum, clearing of lymph nodes around the left gastric artery, and ligation of the left gastric artery were performed. After transecting the stomach for subtotal gastrectomy, reconstruction was performed according to the surgeon’s preference, and tumor location for either gastroduodenostomy or gastrojejunostomy.

#### Firefly

Visible white light was intermittently changed to near-infrared light during lymphadenectomy to visualize ICG fluorescence in the lymphatic chain.

### Perioperative Care

The perioperative protocol was the same in both groups. All patients were encouraged to ambulate on the day of the surgery. The diet consisted of sips of water, a clear liquid diet, and a soft diet on a postoperative day (POD)1, POD2, and POD3, respectively. When no clinical signs of complications, infection, or inflammation were observed, the patients were discharged from the hospital.^18^ Especially, the laboratory parameters including C-reactive protein, body temperature, pulse rate, and neutrophil count on POD 3 were used in the decision-making criteria for discharge after gastrectomy for gastric cancer. Postoperative complications were categorized based on the Clavien-Dindo classification.^19^ The pain was recorded using a visual analog scale in the postanesthesia care unit (30 minutes) and at 1, 2, 4, 6, 12, and 24 hours after the surgery. The daily pain scale score was recorded twice until the patients were discharged from the hospital.

### Outcomes

The primary outcome was the number of retrieved lymph nodes. Secondary outcomes included C-reactive protein (CRP), hospital stay, pain score, body image, and cost. All patients were scheduled for the outpatient department 4 weeks after surgery. Furthermore, patients were monitored for 30 days after surgery for complications. During the outpatient visit, the body image satisfaction questionnaire was administered to patients to evaluate their wound status.^20^ The scale included a 10-item questionnaire on affective, behavioral, and cognitive body image symptoms. Each question assessed body image symptoms from 0 (not at all) to 3 (very much), and the total score ranged from 0 to 30. A higher score indicates a higher level of body image disturbance.

### Sample Size

The initial target sample size for each group was 70. This was derived from previous experience with IRG (n = 80) and CLG (n = 191). The mean number of retrieved lymph nodes was significantly higher in the IRG (53 ± 18.9) than that in the CLG (44.6 ± 16.6). Therefore, to detect the superiority of IRG, 2-sided, with a power of 80% and an alpha error of 5%, presumed with 1:1 matching, 57 patients for each arm were necessary. Overall, 70 patients were included for each component, considering drop-off and matching failures.

### Statistical Analysis

Categorical data were compared using either Pearson’s chi-square or Fisher’s exact test. Normally distributed quantitative data were analyzed using Student’s t-test, and the Mann-Whitney *U* test was used otherwise. All tests were 2-sided with a significance level of 5%. All analyses were performed using Statistical Analysis Software (SAS) (version 9.4; SAS Institute, Cary, NC, USA).

## RESULTS

### Patients

**FIGURE 1.**
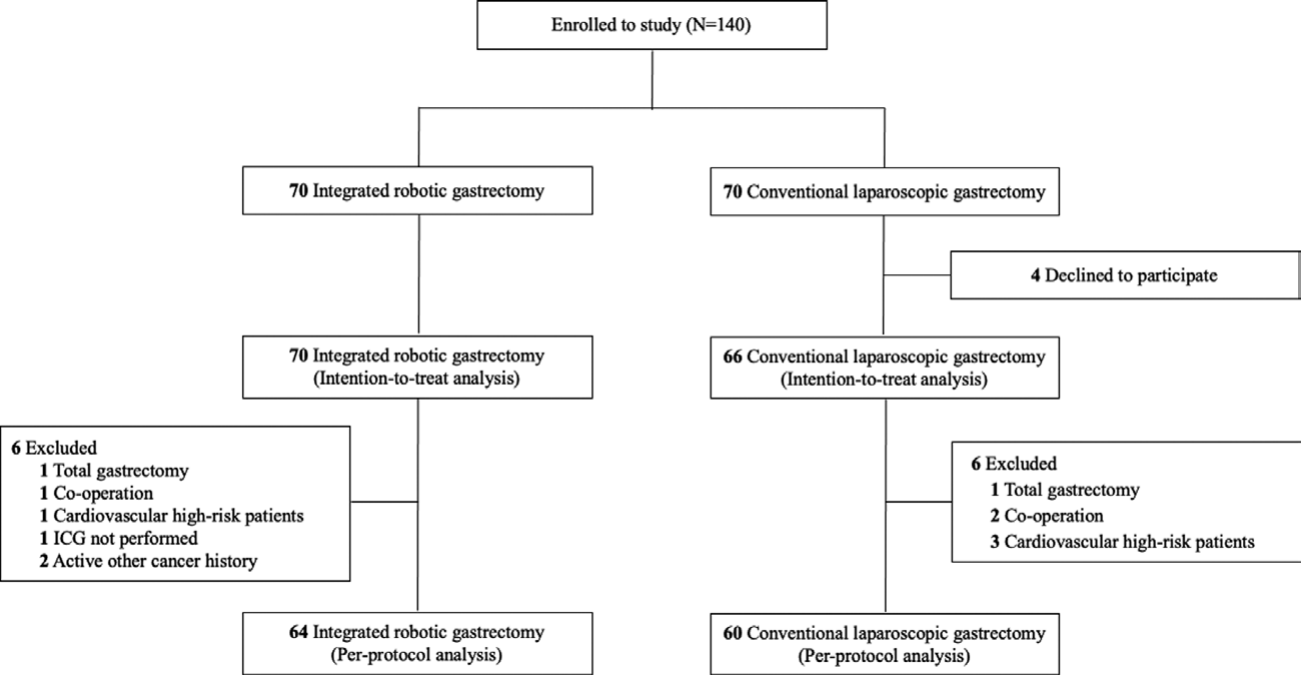
Recruitment and inclusion of patients for the prospective comparative study. ICG, indocyanine green.

Overall, 140 patients were enrolled and included in the IRG (70 patients) and CLG (70 patients) groups between January 2018 and October 2021. Excluding four patients who did not undergo surgery in the CLG group, 136 patients were analyzed for the intention-to-treat dataset (**Figure 1**). In addition, 12 patients were excluded for various reasons. The per-protocol analysis included 124 patients, with 64 and 60 in the IRG and CLG groups, respectively. Clinical features of patients, including age, sex, body mass index, American Society of Anesthesiologists physical status classification, and percentage of previous abdominal surgery, did not differ between the IRG and CLG groups. Furthermore, no statistical differences were observed between the two groups in the pathological T, N, and TNM stages (**Table 1**).

**TABLE 1.** Clinicopathologic Characteristics of Patients

	Intention to Treat			Per Protocol		
	IRG (N = 70)	CLG (N = 66)	*P*	IRG (N = 64)	CLG (N = 60)	*P*
Age, mean (SD), years	58.1 ± 10.7	60.4 ± 11.7	0.230	58.3 ± 10.9	61.0 ± 11.3	0.180
Sex, No. (%)			0.767			0.953
Male	41 (58.6%)	37 (56.1%)		37 (57.8%)	35 (58.3%)	
Female	29 (41.4%)	29 (43.9%)		27 (42.2%)	25 (41.7%)	
BMI, mean (SD), kg/m^2^	24.3 ± 3.1	24.5 ± 3.3	0.717	24.2 ± 2.8	24.5 ± 3.4	0.621
ASA score, No. (%)			0.135			0.285
1	12 (17.1)	8 (12.1)		11 (17.2)	7 (11.7)	
2	45 (64.3)	36 (54.5)		42 (65.6)	36 (60.0)	
3	13 (18.6)	22 (33.3)		11 (17.2)	17 (28.3)	
Previous abdominal surgery, No. (%)	14 (20.0)	11 (16.7)	0.616	13 (20.3)	9 (15.0)	0.439
pT stage, No. (%)			0.926			0.369
pT1	65 (92.9)	61 (92.4)		61 (95.3)	55 (91.7)	
pT2	4 (5.7)	4 (6.1)		2 (3.1)	4 (6.7)	
pT3	0 (0)	1 (1.5)		0 (0)	1 (1.7)	
pT4	1 (1.4)	0 (0)		1 (1.6)	0 (0)	
pN stage, No. (%)			0.681			0.681
pN0	65 (92.9)	64 (97.0)		59 (92.2)	58 (96.7)	
pN1	4 (5.7)	1 (1.5)		4 (6.3)	1 (1.7)	
pN2	1 (1.4)	1 (1.5)		1 (1.6)	1 (1.7)	
TNM stage (8th edition), No. (%)			0.801			0.800
I	68 (97.1)	64 (97.0)		62 (96.9)	58 (96.7)	
II	2 (2.9)	1 (1.5)		2 (3.1)	1 (1.7)	
III	0 (0)	1 (1.5)		0 (0)	1 (1.7)	

Continuous data are expressed as the mean ± standard deviation, and categorical data are expressed as number*s* (%).

ASA indicates American Society of Anesthesiologists; BMI, body mass index; CLG, conventional laparoscopic gastrectomy; IRG, integrated robotic gastrectomy; pN, pathologic lymph node involvement; pT, pathologic depth of invasion; SD, standard deviation; TNM, tumor-node-metastasis.

### Surgical Parameters

**FIGURE 2.**
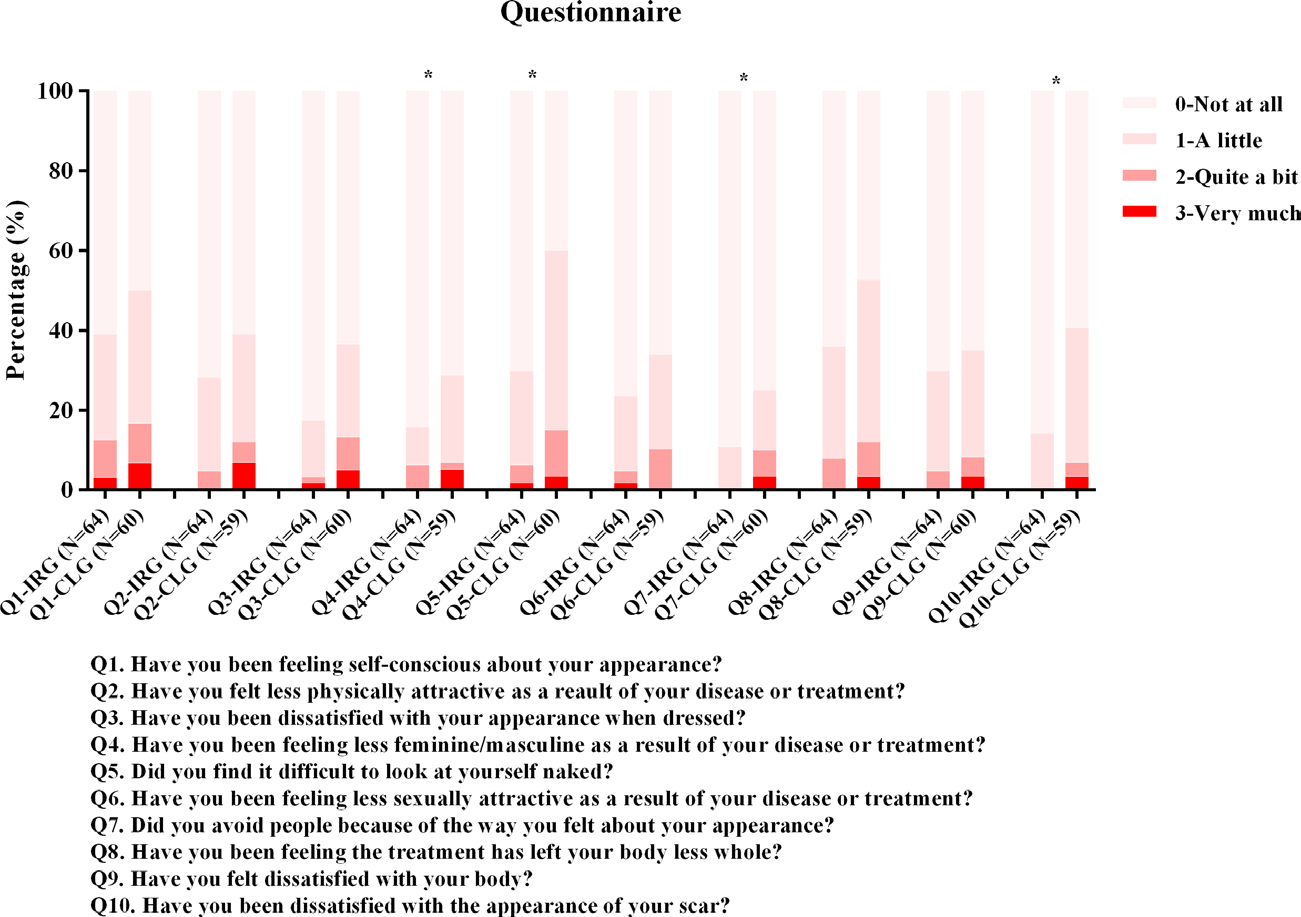
The body image scale for assessing body image dissatisfaction from per-protocol analysis. CLG, conventional laparoscopic gastrectomy; IRG, integrated robotic gastrectomy.

Most surgeries included subtotal gastrectomy and D1+ lymphadenectomy. The IRG group was associated with a shorter hospital stay (*P* < 0.001, Figure S1, http://links.lww.com/AOSO/A231), a shorter time to flatus (*P* = 0.044), and a higher number of retrieved lymph nodes (*P* = 0.016) (**Table 2**). In addition, operation time, estimated blood loss, visual analog scale, and inflammatory markers (such as CRP and white blood cells (WBCs), Figure S2, http://links.lww.com/AOSO/A232) did not differ between the two groups. The amount of drainage was significantly less in IRG group than in CLG group on POD 2 (88.8 ± 72.6 vs 123.0 ± 103.6, *P* = 0.029) but was not obvious in the per-protocol analysis (91.0 ± 74.5 vs 115.1 ± 91.7, *P* = 0.110). The in-hospital and outpatient complication rates did not differ between the two groups (*P* = 0.226 and *P* = 0.702, respectively; **Table 3**). The readmission rate was not significantly different between the two groups (*P* = 0.713 and *P* > 0.999). The body image scale was significantly lower in Q4, Q5, Q7, and Q10, indicating a favorable body image in the IRG group (**Figure 2**). Furthermore, the operation fee and total costs of the IRG group were significantly higher than those of the CLG group (5,574 vs 4,572 USD, *P* < 0.001; 8,276 vs 7,771 USD, *P* = 0.031, respectively; **Table 3**). However, the cost associated with perioperative care was significantly lower in the IRG group (1,750 vs 2,023 USD, *P* = 0.018) but was not obvious in the per-protocol analysis *(P =* 0.055).

**TABLE 2.** Operative Finding

	Intention to Treat			Per Protocol		
	IRG (N = 70)	CLG (N = 66)	*P*	IRG (N = 64)	CLG (N = 60)	*P*
Extent of lymphadenectomy, No. (%)			>0.999			>0.999
D1+	66 (94.3)	63 (95.5)		61 (95.3)	57 (95.0)	
D2	4 (5.7)	3 (4.5)		3 (4.7)	3 (5.0)	
Extent of gastrectomy, No. (%)			>0.999			
Subtotal gastrectomy	69 (98.6)	65 (98.5)		64 (100)	60 (100)	
Total gastrectomy	1 (1.4)	1 (1.5)		0 (0)	0 (0)	
Operation time, mean (SD), min	147.3 ± 28.4	160.0 ± 55.7	0.095	144.0 ± 24.8	154.2 ± 31.9	0.050
Estimated blood loss, mean (SD), ml	38.3 ± 51.4	57.0 ± 84.2	0.118	31.9 ± 35.4	39.3 ± 27.5	0.198
Retrieved lymph nodes, mean (SD), n	41.9 ± 17.4	35.3 ± 14.0	**0.016**	42.1 ± 17.9	35.1 ± 14.6	**0.019**
Time to first flatus, mean (SD), days	2.6 ± 0.6	2.8 ± 0.7	**0.044**	2.6 ± 0.6	2.8 ± 0.7	0.093
Length of hospital stay, mean (SD), days	4.1 ± 1.0	5.5 ± 2.4	**<0.001**	4.1 ± 1.0	5.2 ± 1.8	**<0.001**
Amount of drainage, ml						
POD #0	99.8 ± 45.8	113.0 ± 67.1	0.191	95.1 ± 43.4	112.4 ± 64.6	0.086
POD #1	121.7 ± 108.5	122.5 ± 105.3	0.969	121.6 ± 109.5	117.6 ± 104.1	0.833
POD #2	88.8 ± 72.6	123.0 ± 103.6	**0.029**	91.0 ± 74.5	115.1 ± 91.7	0.110
POD #3	74.6 ± 64.9	108.0 ± 118.7	0.054	77.2 ± 67.4	92.0 ± 88.7	0.341
Visual analog scale mean (SD),						
PACU	4.5 ± 1.3	4.7 ± 1.6	0.432	4.5 ± 1.4	4.7 ± 1.6	0.526
1 hour	3.9 ± 1.3	4.0 ± 1.5	0.590	3.9 ± 1.3	4.1 ± 1.6	0.537
2 hours	3.0 ± 1.1	3.2 ± 1.1	0.421	3.0 ± 1.1	3.2 ± 1.0	0.601
4 hours	2.9 ± 0.8	2.8 ± 0.8	0.684	2.8 ± 0.8	2.8 ± 0.8	0.850
6 hours	3.2 ± 1.2	3.0 ± 1.2	0.359	3.1 ± 1.2	3.0 ± 1.2	0.414
12 hours	2.9 ± 1.1	2.8 ± 0.9	0.628	2.8 ± 1.1	2.7 ± 0.9	0.601
24 hours	2.4 ± 0.9	2.7 ± 0.9	0.122	2.4 ± 0.9	2.6 ± 0.8	0.404
48 hours	1.8 ± 0.8	1.8 ± 0.7	0.971	1.8 ± 0.6	1.8 ± 0.7	0.882

Continuous data are expressed as the mean ± standard deviation, and categorical data are expressed as number*s* (%).

IRG, integrated robotic gastrectomy; CLG, conventional laparoscopic gastrectomy; POD, postoperative day; PACU, postanesthesia care unit; SD, standard deviation

**TABLE 3.** Postoperative complication and cost analysis

	Intention to treat			Per protocol		
	IRG (N = 70)	CLG (N = 66)	*P*	IRG (N = 64)	CLG (N = 60)	*P*
In-hospital complication, No. (%)			0.266			0.349
Grade 1	12 (17.1)	17 (25.8)		11 (17.2)	16 (26.7)	
Grade 2	9 (12.9)	9 (13.6)		9 (14.1)	9 (15.0)	
Grade 3^a^	0 (0)	2 (3.0)		0 (0)	1 (1.7)^b^	
Outpatient complication, No. (%)			0.702			>0.999
Grade 1	0 (0)	1 (1.5)		0 (0)	0 (0)	
Grade 2	2 (2.9)	1 (1.5)		1 (1.6)	0 (0)	
Grade 3^c^	1 (1.4)	2 (3.0)		1 (1.6)	1 (1.7)^d^	
Readmission	3 (4.3)	4 (6.1)	0.713	2 (3.1)	1 (1.7)	>0.999
Hospital stay cost, Won	1,355,388 ± 655,886 (952 ± 461 USD)	1,531,250 ± 661,805 (1,076 ± 465 USD)	0.122	1,332,699 ± 640,323 (937 ± 450 USD)	1,493,632 ± 636,705 (1,049 ± 447 USD)	0.163
Operation fee, Won	7,931,634 ± 765,117 (5,574 ± 538 USD)	6,648,877 ± 1,064,690 (4,572 ± 748 USD)	**<0.001**	7,848,042 ± 468,862 (5,515 ± 329 USD)	6,554,394 ± 903,454 (4,606 ± 635 USD)	**<0.001**
Perioperative care, Won	2,490,533 ± 442,751 (1,750 ± 311 USD)	2,878,918 ± 1,227,794 (2,023 ± 863 USD)	**0.018**	2,474,564 ± 438,539 (1,739 ± 308 USD)	2,736,145 ± 981,681 (1,923 ± 690 USD)	0.055
Total cost, Won	11,777,555 ± 1,134,383 (8,276 ± 797 USD)	11,059,045 ± 2,425,836 (7,771 ± 1705 USD)	**0.031**	11,655,304 ± 957,680 (8,190 ± 673 USD)	10,784,171 ± 1,914,186 (7,578 ± 1345 USD)	**0.002**

Categorical data are expressed as numbers (%)

IRG, integrated robotic gastrectomy; CLG, conventional laparoscopic gastrectomy

^a^Details of grade 3 in-hospital complications are as follows: pigtail insertion due to intra-abdominal fluid collection and

^b^re-operation due to postoperative obstruction

^c^Details of grade 3 outpatient complications are as follows: for the integrated robotic group, pigtail insertion due to intra-abdominal fluid collection; for the conventional laparoscopic group, pigtail insertion due to intra-abdominal fluid collection; and

^d^endoscopic hemostasis due to upper gastrointestinal bleeding.

## DISCUSSION

This is the first prospective trial to evaluate the clinical benefits of robotic technology, integrated with a two-port platform and fluorescence-guided system, compared with conventional laparoscopic gastrectomy. The surgical outcomes clearly show the value of the IRG group: a significantly higher number of retrieved lymph nodes (41.9 vs 35.3), shorter hospital stay (4.1 vs 5.5), a shorter time to flatus (2.6 vs 2.8), better cosmetic effect, and lower cost for perioperative care, which is marginally significant. However, no significant difference was observed between the two groups regarding complication rate, operation time, intraoperative bleeding, postoperative inflammatory reaction, or pain score.

Currently, no gold standard exists for the priority of short-term perioperative parameters. Complications, number of retrieved lymph nodes, hospital stay, operation time, intraoperative bleeding, pain, cosmesis, and cost were considered relevant parameters. Among them, the number of retrieved lymph nodes was chosen as the primary endpoint to confirm enhanced lymphadenectomy using fluorescence without being jeopardized by two-port surgery. Previously, a similar retrospective study conducted by our group showed a significantly higher number of retrieved lymph nodes in the IRG group.^16^ This study confirmed the previous findings prospectively. Fluorescence lymphography enables surgeons to perform en bloc resection of lymph nodes without breakage, preventing injury to the surrounding tissue and consequently speeding up the surgical procedure.^14^ It could be worried that fluorescence-guided surgery forces surgeons to resect everything visualized, causing unnecessary injury. However, easy identification of lymphatic chains between the normal tissues can decrease the risk of pancreatic injury and associated time-consuming procedures to fix the problems encountered during the surgery. It should be validated in further study. The majority of patients enrolled in this study had early cancer lesions that oncological benefits by better retrieval of lymph nodes are not crucial. However, this feature encourages surgeons to use IRG for the surgery of advanced gastric cancer. In addition to the enhanced visualization using Firefly, the Single-site system enabled surgeons to perform two-port gastrectomy, appreciating the wrist function of the robotic arm and console system. Furthermore, it enables surgeons to perform suprapancreatic lymphadenectomy easily retracting the pancreas or surrounding tissues, and provides a better operative environment during two-port gastrectomy. Integrating both fluorescence-guided surgery and reduced-port approach, we moved one step forward to the ideal surgery, which is oncologically radical surgery without a scar.

This trial showed the superior short-term clinical advantage, specifically a shorter hospital stay. Despite the theoretical benefit of reduced-port surgery, previous reports have revealed no difference in the hospital stay in the reduced-port group.^16,21–23^ Therefore, we speculate that two factors contribute to early recovery following IRG. First, the surgical procedures were optimized using accumulated experience, and unnecessary time wastage was avoided; subsequently, the operation time was shortened. As previously reported, a critical drawback of robotic surgery is the longer operation time.^24,25^ Particularly, a longer operation time is associated with higher inflammatory laboratory value, pain, and a more extended hospital stay.^26^ As previously shown in an analysis of consumed time for instrument changes,^27^ time wastage while changing the robotic arm is significant. Therefore, our series used an auto-load laparoscopic clip rather than a robotic clip during IRG to save operative time. The second reason is that we selected the patients most likely to benefit from two-port gastrectomy. This study included patients with EGC who underwent subtotal gastrectomy with D1+ lymph node dissection. If the pain associated with surgical stress is the sum of visceral and somatic pain caused by gastric resection and trocar site incision, then reduced-port surgery would lower pain felt by the patient only when visceral pain is sufficiently small. In contrast, patients with advanced gastric cancer undergoing D2 lymph node dissection or total gastrectomy require an extensive surgical approach and a long operation time. In such cases, reduced-port surgery is useless or may be harmful to minimize surgical stress.

IRG showed similar complications, operation time, intraoperative bleeding, and pain in this study. Contrary to our hypothesis, the pain scale in the IRG group was not significantly different from that in the CLG group. Although the IRG showed less pain during the first 2 hours, the difference was not significant. It appears the same was true for laboratory values of the inflammatory parameters, such as CRP and WBC values. However, the laboratory values at POD5, which do not represent all patients in the IRG group, should be considered since more than half of the IRG was discharged at POD5, and the laboratory value of IRG at POD5 was from patients with late recovery (n = 16).

Cosmetic effects have long been neglected in the era of oncologic surgery. However, if long-term survival is anticipated, particularly in young patients, scarring and associated body self-image could be relevant in selecting the type of surgery. This study showed better satisfaction in Q4, Q5, Q7, and Q10, which cannot be neglected in young patients.

The robotic approach’s shortcoming includes its high costs compared with laparoscopic surgery.^11^ A previous multicenter prospective study in Korea reported that the total cost of robotic surgery was 6.4 M KRW (4490 USD) more expensive than that of laparoscopic surgery.^8^ In this study, the cost difference was smaller than the previous multicenter prospective study (1.4M KRW (1000 USD)). The cost difference between IRG and CLG analyzed in this study is significantly different (*P* = 0.031) but financially negligible. In detail, the operation fee of IRG is still expensive but close to that of CLG; the other cost is cheaper in IRG. The robotic arm of a Single-site system is cheaper than a conventional robotic arm contributing to the low cost of the robot in this study. In addition, a shorter hospital stay in IRG contributed to decreased perioperative costs although they did not reach a significant difference. Finally, robotic surgery provides a clear but overlooked financial advantage. Through the operative procedure, only a surgeon and one assistant are required to complete the entire procedure in the IRG, compared with the CLG, which requires a surgeon and two assistants. This is particularly important in institutions or countries lacking surgical assistance. It will be revealed if detailed cost accounting is performed, including expense analysis for human resource costs. Hence, cost differences would not be an issue for robotic surgery in the future since the high cost of laparoscopic surgery compared with open surgery is no longer a limitation today.

This study’s major limitation was the generalization of the results. Single-surgeon experience funded by the company making surgical robots could be subject to bias during data analysis and interpretation, although we conducted a sound clinical trial. However, this evidence will pave the path for the application of two-port robotic surgery with fluorescence-guided surgery. In addition, several publications regarding this IRG demonstrate the scientific implementation of new surgical procedures.^15,26,28–33^ Beginning with a clinical trial for feasibility and safety,^15^ IRG has been attempted in clinical practice for several years with good clinical outcomes.^16^ Finally, this prospective comparative study will prove the robustness of the IRG procedure.

In conclusion, the IRG group had a higher number of retrieved lymph nodes, a shorter hospital stay, and a better body image scale than those of the CLG group. Evidently, robotic technology with additional features of fluorescent lymphography can maximize radicality, while the two-port approach can minimize the invasiveness of surgery.

## ACKNOWLEDGMENTS

We thank the patients, the investigators and their teams who took part in this study.

## Supplementary Material


