# Supplementary figures and images for: Fluorescence-guided Two-port Robotic Gastrectomy Versus Conventional Laparoscopic Gastrectomy: A Nonrandomized Controlled Trial
Source: Ann Surg Open. 2023 Jul 26;4(3):e318. doi: 10.1097/AS9.0000000000000318 (PMC10513269; doi:10.1097/AS9.0000000000000318)

### Hospital stay

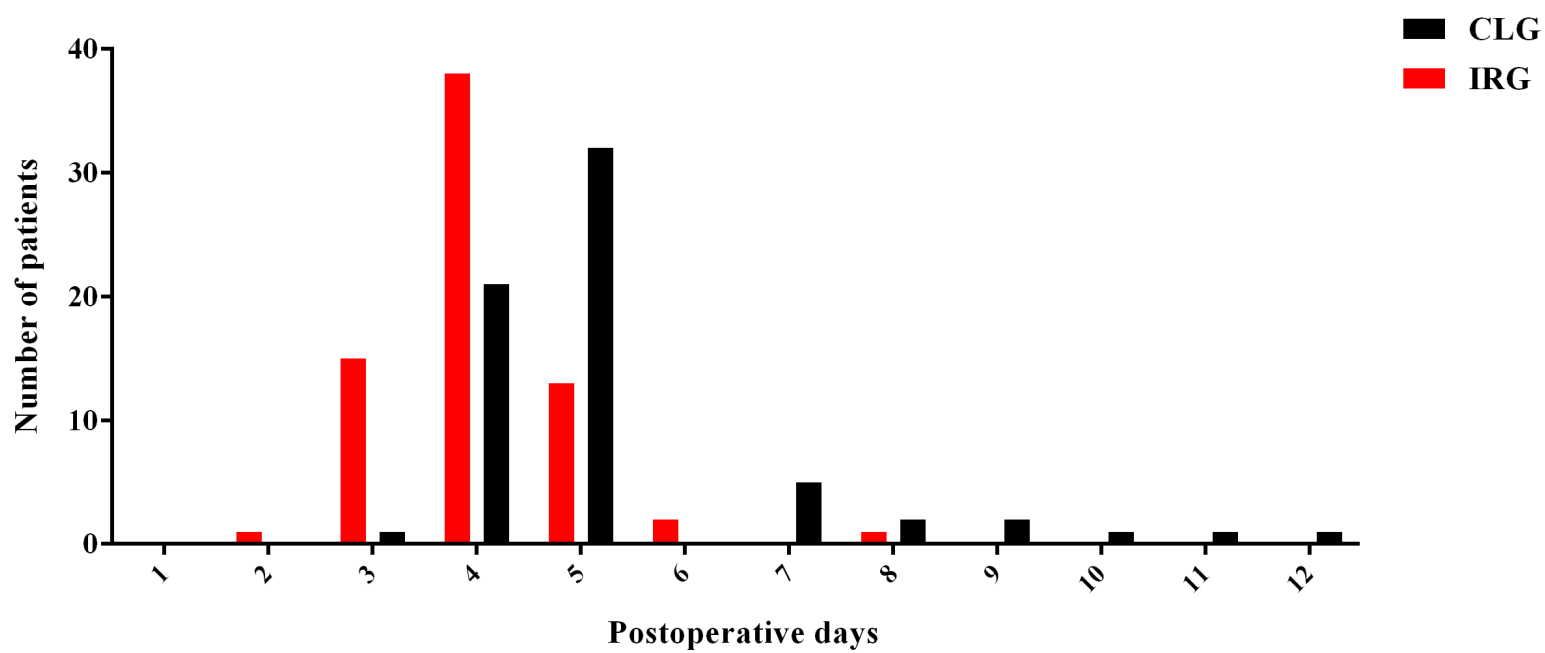

Supplement: Supplementary file 1 [file as9-4-e318-s001.pdf]

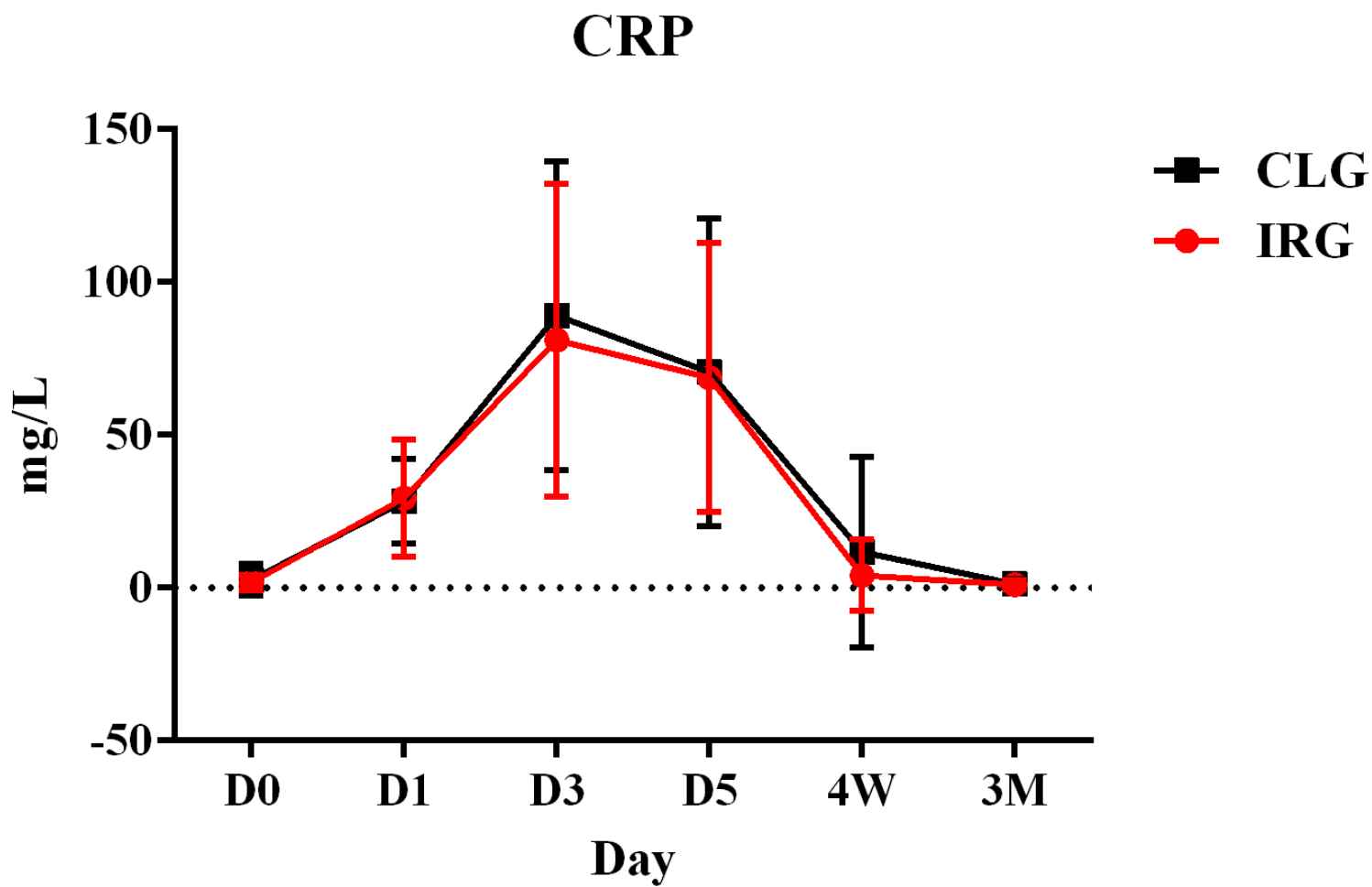

Number of patients

|     |    |    |    |    |    |    |
|-----|----|----|----|----|----|----|
| CLG | 66 | 66 | 66 | 43 | 66 | 66 |
| IRG | 70 | 70 | 69 | 16 | 70 | 70 |

## WBC

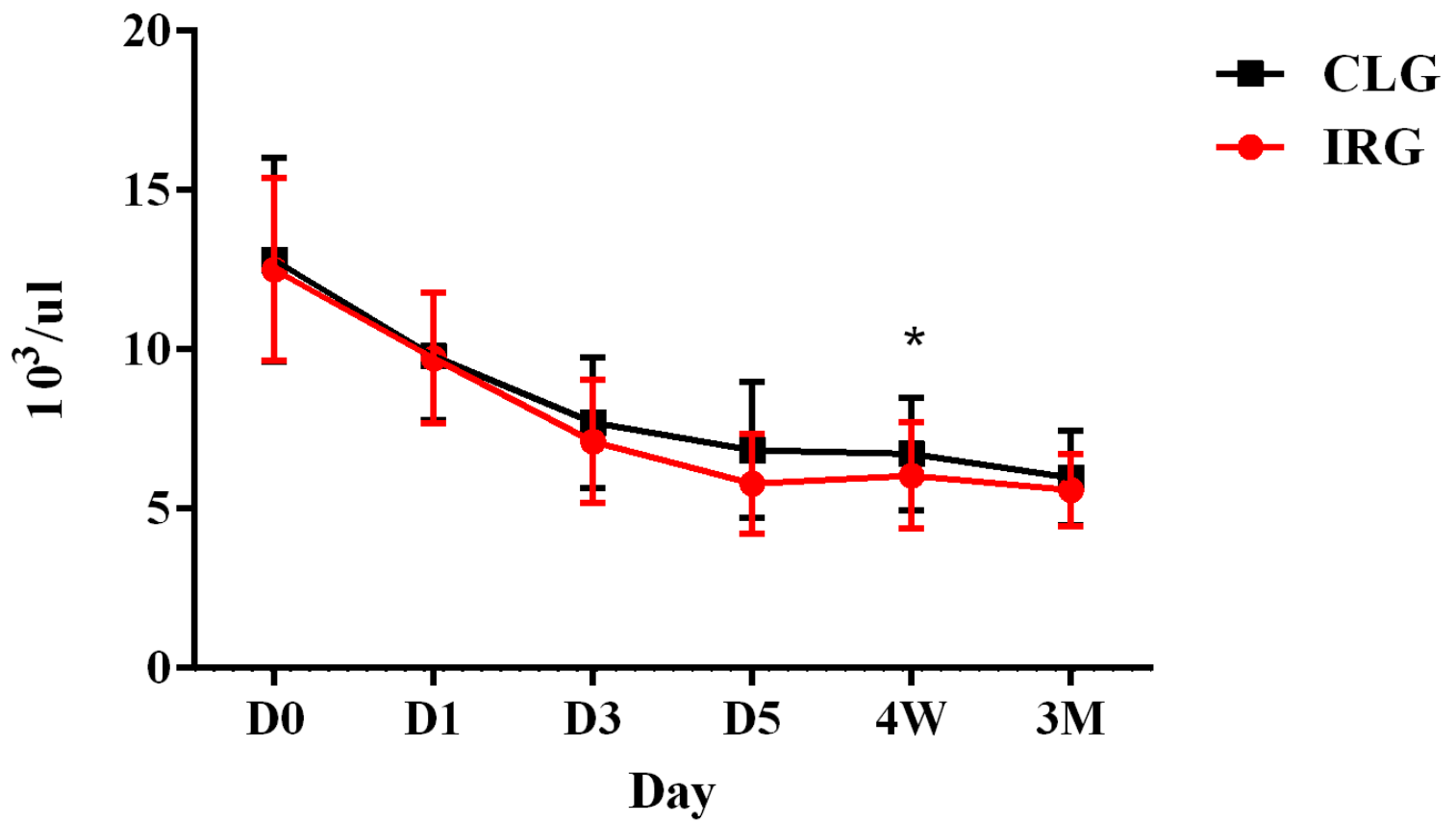

Number of patients

|     |    |    |    |    |    |    |
|-----|----|----|----|----|----|----|
| CLG | 66 | 66 | 66 | 43 | 66 | 66 |
| IRG | 70 | 70 | 69 | 16 | 70 | 70 |

Supplement: Supplementary file 2 [file as9-4-e318-s002.pdf]
